# Supplementary material for: Genetic comparisons of interleukin-17 reveal a framework for complex signaling evolution
Source: bioRxiv. 2026 Apr 14:2026.04.13.718218. Preprint. [Version 1] doi: 10.64898/2026.04.13.718218 (PMC13105070; doi:10.64898/2026.04.13.718218)
Supplement: Supplement 3 [file media-3.zip › Supplemental files for paper/ERC/IL17xIL17_comparisons/IL17RD_IL17REL.pdf]

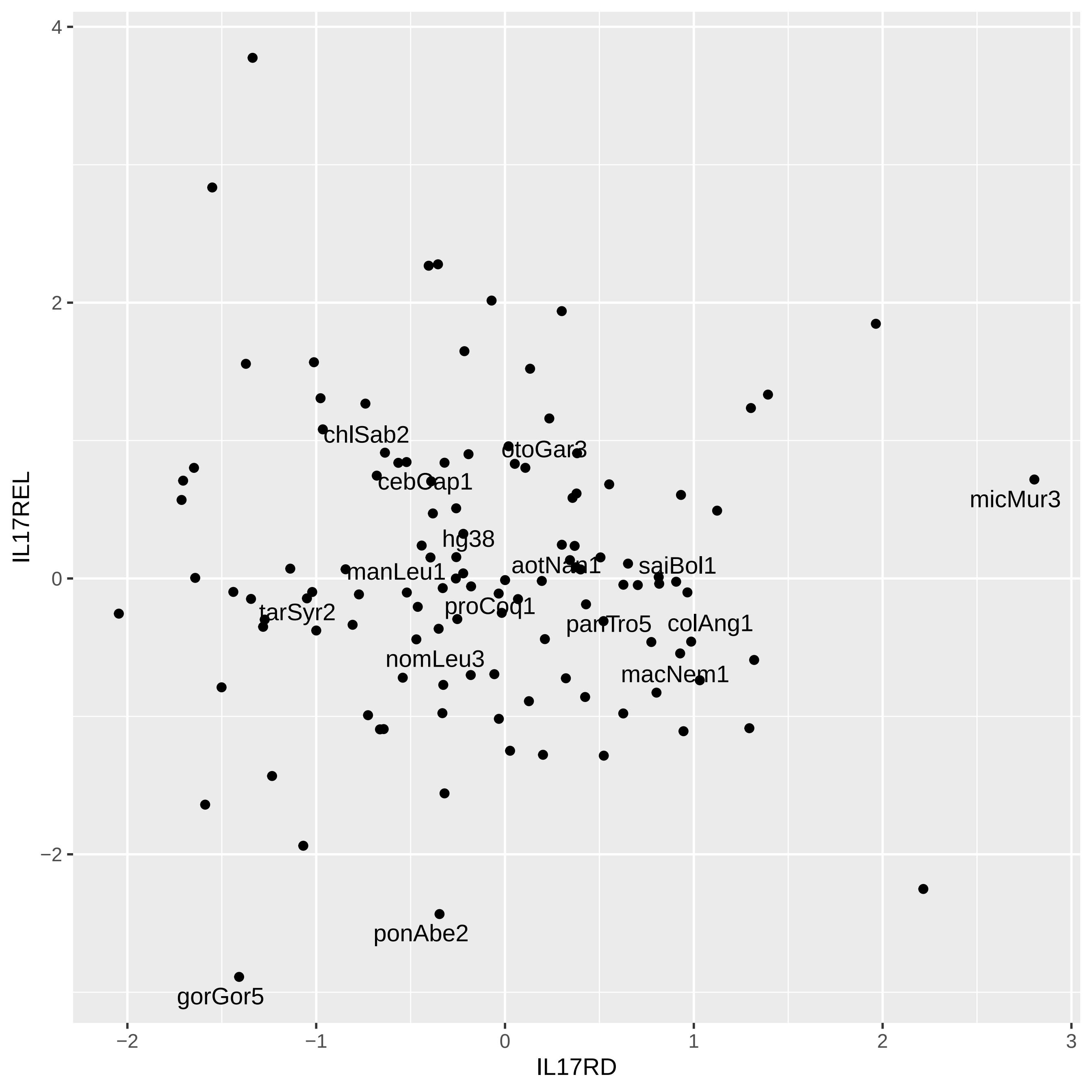

4
2
0
−2
−2
−1
0
1
2
3
IL17REL
IL17RD
chlSab2
otoGar3
cebCap1
micMur3
hg38
aotNan1
saiBol1
manLeu1
proCoq1
tarSyr2
panTro5
colAng1
nomLeu3
macNem1
ponAbe2
gorGor5
